# A dataset for connecting similar past and present causalities

**DOI:** 10.1016/j.dib.2020.105185

**Published:** 2020-01-27

**Authors:** Ryohei Ikejiri, Yasunobu Sumikawa

**Affiliations:** a The University of Tokyo, Japan; b Tokyo Metropolitan University, Japan

**Keywords:** Digital history, Event category, Text classification, Temporal classification, Information retrieval

## Abstract

In this data article, we present a dataset that includes past causalities and categories to connect similar past and present causalities. First, we collect past causalities by referencing certain well-known Japanese high-school textbooks. Subsequently, we select 138 causalities that are useful for analogizing from the causalities to considering solutions for confront present social issues. To enhance the analogy, we describe each causality in three contexts: background including problems, solution methods, and their results. We define 13 categories based on the selected causalities and Encyclopedia of Historiography. The past causalities belong to more than one category. In addition, to train machine learning models including classifier, we collect 900 past events from Wikipedia, and assign one or more categories to the past event data. We perform statistical analyses to understand the quality of the dataset. The proposed applications of the dataset include training machine learning models such as classifiers for past causalities and information retrieval for ranking present social issues according to the similarities between the present and past causalities.

**Table:** Specifications Table

Subject	*Information Systems*
Specific subject area	*Data mining, Digital History, Labeled Dataset for Machine Learning*
Type of data	Table.
How data were acquired	Past causalities described by authors by referencing past causalities in some Japanese Textbooks for World History Past events are crawled from Wikipedia
Data format	Filtered Raw data of Fig. 1, Fig. 2, Fig. 3, Fig. 4, Fig. 5, Fig. 6 and Table 4 are stored in: causality_regional_distribution.tsv (Fig. 1), causality_temporal_distribution.tsv (Fig. 2), and Statistics.tsv (Fig. 3, Fig. 4, Fig. 5, Fig. 6 and Table 4).
Parameters for data collection	
Description of data collection	The collection processes were performed by manual inspections of experts who have Ph. D. degrees in related research fields.
Data source location	As for describing data of past causalities, we referenced past causalities in Japanese textbooks: *Shosetu Sekaishi B* (Se B 304) [1] and *Sekaishi B* (Se B 301) [2]. As for data collected Wikipedia texts, we used following links: https://www.en.wikipedia/* where “*” is replaced by one of from 1 to 1999.
Data accessibility	Repository name: Zenodo Data identification number: zenodo.3,601,707 Direct URL to data: https://doi.org/10.5281/zenodo.3601707
Related research article	*Ikejiri, R., Sumikawa, Y.: Developing world history lessons to foster authentic social participation by searching for historical causation in relation to current issues dominating the news. Journal of Educational Research on Social Studies 84, 37–48 (2016). (in Japanese).*https://doi.org/10.20799/jerasskenkyu.84.0_37

**Table:** 

**Value of the Data** • The dataset is useful for training machine learning models as a labeled dataset to predict causality—causality similarity over time. • The dataset can be beneficial for HistoInformatics researchers and history education researchers who are developing new teaching tools to bridge the gap between the past and present, and computer scientists working on temporal data mining including information retrieval. • Once event detections and classifiers are developed by the dataset, they can use them to assign causality categories to other new data. Through this process, the dataset is able to re-examine how accurately categories are defined, identify which categories are (in) dependent of time, or add new categories to the dataset. • We provide the scores of statistical analyses to measure/evaluate dataset quality for estimating which machine learning models can be effectively trained on our dataset. In addition, from these scores, it is able to develop other dataset such as translation to Chinese, adding new temporal events with detail comparisons between our scores and new dataset to identify changes of accuracies.

## 1 Data Description

The published dataset [3] (see metadata in Table 1) consists of seven types of data. The first type includes 138 past causalities in historical_causalities_data.csv file. In this dataset, all causalities include their backgrounds and results. The second type includes the categories that are defined for causalities stored in historical_causalities_categories.csv. This file includes 13 categories: **Reign (Rg)**, **Diplomacy (Dp)**, **War (Wr)**, **Production (Pr)**, **Commerce (Cr)**, **Study (St)**, **Religion (Rl)**, **Literature and Thought (LT)**, **Technology (Tc)**, **Popular Movement (PM)**, **Community (Cn), Disparity (Ds)** and **Environment (En).** The third type includes 900 past event data to support training machine learning models. In the dataset, all events include only descriptions of the events; in other words, the event data excludes both backgrounds and results of the events. The past_events_wikipedia.tsv file contains the descriptions of the 900 past events and their categories. The three files (historical_causalities_regions.tsv, causality_regional_distribution.tsv, and causality_temporal_distribution.tsv) provide additional information of the causalities included in the historical_causalities_data.tsv file. The historical_causalities_regions.tsv includes region where each causality occurred. The other two files (causality_regional_distribution.tsv and causality_temporal_distribution.tsv) include distributions of causalities by regions and centuries, respectively. Last, Statistics.tsv file includes scores of statistical analyses, which are described in “**Statistical Analysis**” section. This file provides all raw scores for estimating which machine learning models are useful for several kinds of applications, e.g., classification and information retrieval (IR) algorithms that can bridge the past and present.

**Table 1:** Database files.

File name	Content	Columns
historical_causalities_data.tsv	Detail of stored causalities.	Causality ID: IDs for causalities. Century: Centuries when causalities occurred. BC centuries are represented with minus (”-”) in this file. Title: Names of the causalities. Content: Descriptions of the causalities.
historical_causalities_regions.tsv	Regions where the causalities occurred.	Causality ID: IDs for causalities. Regions: Related regions.
past_events_wikipedia.tsv	Descriptions of past events	Year: A year of Wikipedia article titles that include the past event Categories: Names of categories assigned to the past event. Text: Descriptions of the past event.
historical_causalities_categories.tsv	Categories of the causalities.	Causality ID: IDs for causalities. Categories: Names of categories.
causality_regional_distribution.tsv	Numbers of causalities for all regions.	The first column includes centuries. BC centuries are represented with minus (”-”) in this file.The second column includes the number of causalities when occurred in the century of the raw.
causality_temporal_distribution.tsv	Numbers of causalities for all centuries.	The first column includes region names. The second column includes the number of causalities where occurred in the region of the raw.
Statistics.tsv	Scores of statistical analyses described in this paper	This file contains all scores of statistical analyses described in the “**Statistical Analysis**” section. This file provides raw data of Fig. 3, Fig. 4, Fig. 5, Fig. 6.

**Table 2:** Statistics of the whole dataset published in this paper.

Number of categories	13
Number of historical causalities	138
Number of past events	900
Ave. Num. of causalities per category	10.6

**Table 3:** Numbers of the causalities and past events in each category.

	Rg	Dp	Wr	Pr	Cr	St	Rl
Num. of Causalities	46	64	29	29	51	18	24
Num. of Past Events	405	229	370	7	25	33	183

	LT	Tc	PM	Cn	Ds	En
Num. of Causalities	26	17	23	49	22	20
Num. of Past Events	40	31	70	32	14	32

**Table 4:** Intra-category Meta-data and TF-IDF + JS similarities.

	Rg	Dp	Wr	Pr	Cr	St	Rl
Meta-data	10.6%	12.0%	14.1%	12.4%	13.6%	12.4%	13.2%
MI	0.1395	0.1422	0.1478	0.1543	0.1433	0.1473	0.1650
Jaccard	0.0175	0.0179	0.0176	0.0181	0.0176	0.0187	0.0180
TF-IDF + JS	0.9575	0.9557	0.9530	0.9516	0.9540	0.9603	0.9499
	LT	Tc	PM	Cn	Ds	En	*Total*
Meta-data	12.4%	12.2%	13.5%	11.5%	12.5%	12.8%	*12.5%*
MI	0.1486	0.1396	0.1447	0.1394	0.1401	0.1511	*0.1463*
Jaccard	0.0184	0.0186	0.1769	0.0186	0.1837	0.0185	*0.0552*
TF-IDF + JS	0.9536	0.9584	0.9546	0.9593	0.9488	0.9540	*0.9546*

## 2 Experimental design, materials, and methods

### Causality Data Collections

The 138 past causalities were created by authors in three steps. First, we collected over 700 past causalities by referencing well-known Japanese high-school textbooks: Shosetu Sekaishi B (Se B 304) [1] and Sekaishi B (Se B 301) [2]. Second, we selected the causalities if they could be useful for considering solutions for present social issues. Finally, we described each causality in three contexts: background including problems, solution methods, and their results.

### Category Definition

The causality categories are defined to organize the causalities with the useable historical framework [5] as described in Ref. [4]. In Ref. [5], Lee claims that causalities over different times can be bridged if they belong to the framework because the framework is an overview of the long-term patterns of change and not a mere outline story skimming a few peaks of the past. Under this idea, the category definition processes comprised 2 steps. First, we reviewed Encyclopedia of Historiography [6] to define categories for connecting past and present causalities. In the review process, we listed all the main topics from the encyclopedia and subsequently selected a topic only if it included the long-term patterns of change. Second, we evaluated if each topic included causalities independent of time. As we extracted causalities from history textbooks, we divided them into three temporal periods: ancient, medieval and modern periods. If a topic included causalities from the three temporal periods, we used it as a category in the dataset. Moreover, we added some new categories in the dataset if we found new topics that included causalities from the three temporal periods. Finally, we defined the 13 categories described in **Data Description** section.

### Event Data Collections

The 900 past event data were crawled from Wikipedia articles whose tiles were the years from 1 to 1999, for example, http://en.wikipedia.org/1. The collection process was as follows: 1) All events were crawled from yearly Wikipedia articles. 2) It was manually reviewed whether the crawled events could be useful to consider any solutions for present social issues. 3) At most, 50 events per century were randomly sampled to cover a wide range of durations.

### Basic Statistics

Table 2, Table 3 summarize the statistics of the entire published dataset and the number of causalities for each category, respectively.

**Fig. 1:**
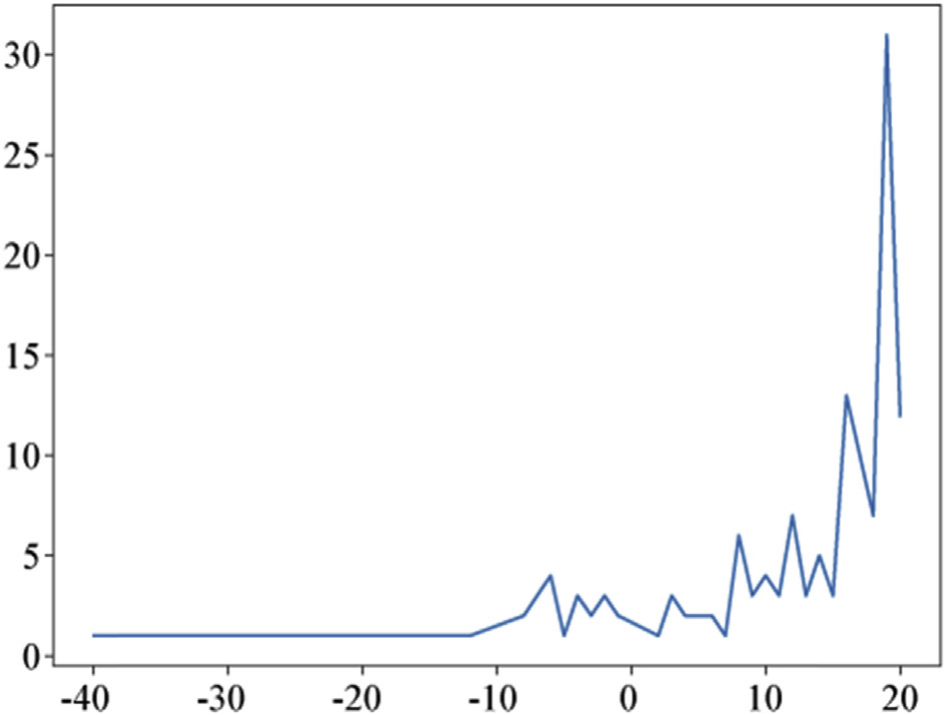
Numbers of causalities per century. This figure represents B. C. years as hyphen (”-”).

**Fig. 2:**
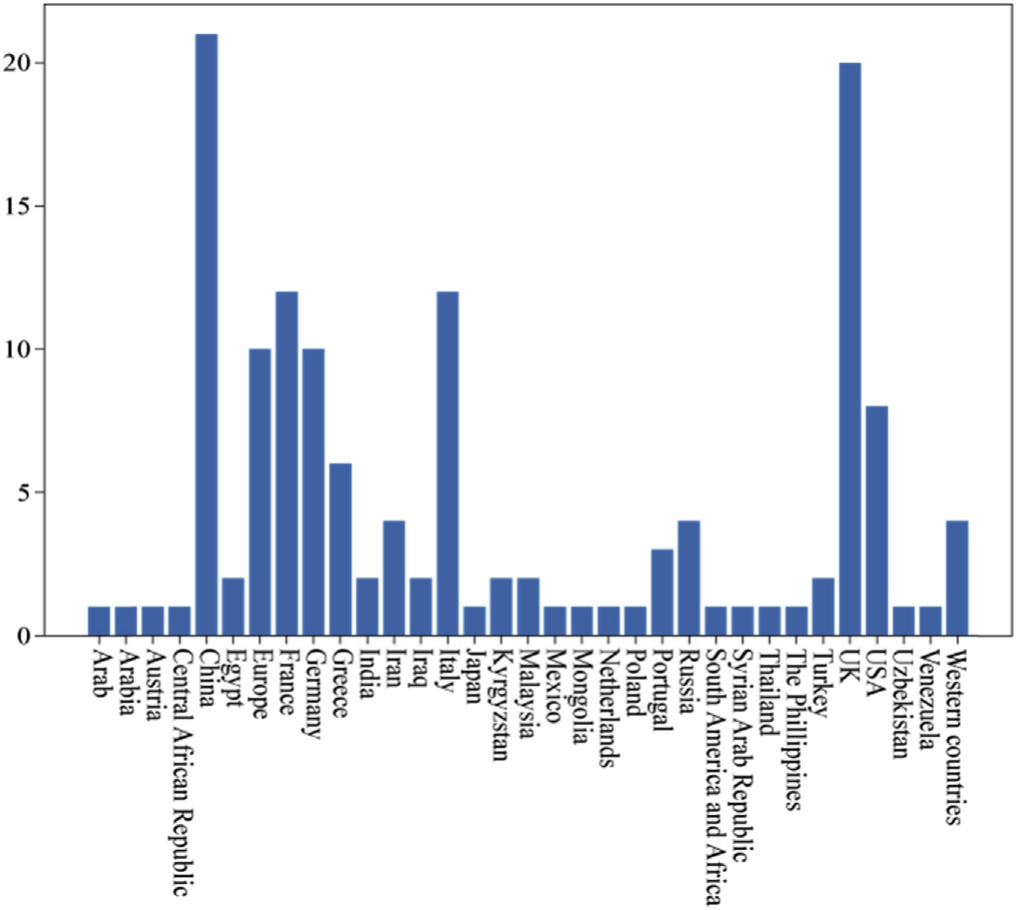
Numbers of causalities per country/region.

Fig. 1, Fig. 2 plot distribution of the numbers of causalities by region and centuries. These figures help us to understand tendency of the published dataset because temporal and spatial features are the most important features of history. Fig. 1 plots the number of causalities per century. Naturally, the distribution curve increases near the present. This indicates that the closer the causalities are located to the present on the temporal axis, the greater is their usability for considering solutions for present issues. Fig. 2 plots the distribution of the number of causalities where they occurred. We can see that most causalities occurred in China and Europe, as they have long-term histories.

### Statistical Analysis

In addition to the basic statistics, the published dataset provides scores of similarity between data points and statistics of clusters to help to train machine learning models. The provided scores are results of the following five analyses.

1. Calinski and Harabasz (CH) [7]. This measure estimates how close all data to each other in a cluster and how far data in different clusters locate. Thus, the higher score of this measure indicates the high quality of the given clusters. The formal equation is defined as follows:

(1) $$CH\left(k\right)=\;\frac{\left(n-k\right)\;B\left(k\right)}{\left(k-1\right)\;W\left(k\right)}$$

where *B*(*k*) and *W*(*k*) are intra- and inter-cluster sums of squares for *k* clusters, respectively, and *n* is the number of clustered data.

2. Mutual information (MI). This measure evaluates the similarity of two categories A and B as an information-theoretic approach. Let *P*(*a*) and *P*(*b*) are marginal probabilities, and *P*(*a, b*) is a joint probability. MI calculates volumes of information a given set generates about the other set. This is done by the following equation.

$$MI\left(A,\;B\right)=\;\sum_{b\in B}\sum_{a\in A}P\left(a,\;b\right)\log\frac{P\left(a,\;b\right)}{P\left(a\right)\;P\left(b\right)}$$

The provided scores of this dataset are generated from adjusted MI (AMI) [8] that is a variant of MI defined as follows:

$$AMI\left(A,\;B\right)=\;\frac{MI\left(A,\;B\right)-E\left(MI\left(A,\;B\right)\right)}{max\left(H\left(A\right),\;H\left(B\right)\right)-E\left(MI\left(A,\;B\right)\right)}$$

where E(MI(A,B)) is the expected MI between two given categories, max is a function that returns the largest value among given values, and H(A) is the entropy of category A.

3. Jaccard index (Jaccard). This measure employs an assumption that if two sets $S_{A}$ and $S_{B}$ for two categories A and B have many common data, then the two sets are similar to each other. As the size of given sets affects the common numbers, this measure normalizes the score by taking account of the total sizes of given two sets. In other words, if a given set is huge compared with other sets, the huge set tends to include several elements of other sets. This idea is represented as follows:

$$Jaccard\left(S_{A},S_{B}\right)=\frac{\left|S_{A}\cap S_{B}\right|}{\left|S_{A}\cup S_{B}\right|}$$

4. TF-IDF + Jensen–Shannon (JS) divergence. This is an entropy-based measurement; the lower this score, the more similar the two given probability distributions are. As our dataset includes texts written in natural language, we first apply TF-IDF to convert the text into numbers. Let w, d and W(d) are a word, a document, and a word set of d. The TF-IDF estimates the importance of w in d by counting the numbers of occurrences of the word in the document and by the numbers of documents including the word. Once all data can be converted to vectors whose elements are scores of the importance of the words, the similarity between two data can be measured by JS divergence that is an extension of Kullback—Leibler (KL) divergence. These approaches are defined as follows:

$$TF-IDF\left(w,\;d\right)=\;tf_{w,\;\;d}\frac{\left|D\right|}{\left|\left\{d^{'}\in D\;\text{|}\;w_{i}\in W\left(d'\right)\right\}\right|}$$

$$KL\left(A,\;B\right)=\;\sum_{a\in A,\;b\in B}a\;\log\frac{a}{b}\;\;$$

$$JS\left(A,\;B\right)=\;\frac{1}{2}KL\left(A,\;M\right)+\;\frac{1}{2}KL\left(M,\;B\right)$$

where M is $\frac{1}{2}\left(A+B\right)$.

5. Meta-data similarity. This measure counts the number of common categories shared by two causalities. Similar to the Jaccard index, the higher the score, the more similar the two causalities are. Thus, given two causalities, the measure is represented as the sum of the common categories. This is formally defined as follows:

$$Meta\left(FV_{i},\;FV_{j}\right)=\sum_{k=1}^{FV}AND\left(FV_{ik},\;FV_{jk}\right)$$

where *AND* is the logical AND. It is 1 if both operands are 1; otherwise, it is 0. This measure considers the feature vectors ($FV_{i}$ and $FV_{j}$) of two causalities that are defined from the categories of the two causalities. If causality $C_{i}$ has the *k*th category, then $FV_{ik}$ is 1; otherwise, it is 0.

We applied the above measures for all combinations of causalities within each category (intra-category) and within two different categories (inter-category), which are described as follows:

1. Intra-category Similarity. This similarity represents the average score of similarity between all combinations of two causalities in a category.
2. Inter-category Similarity. This similarity represents the average score of similarity between all combinations of two causalities from two different categories.

### Scores of Statistical Analysis

The CH score for the past causality data was 1.0829. This indicates that the intra-cluster and inter-cluster sums of squares for the *k* clusters are almost the same. Table 4 shows all scores of all intra-category measurements. Overall, these scores indicate that all texts in the same category are not similar to each other. These scores indicate that the published dataset covers several kinds of causality topics. If it is necessary to train machine learning models only on the causality texts, it is better to use simple IR algorithms, for example, the query word matching method, or to employ transfer learning using the categorized past events.

**Fig. 3:**
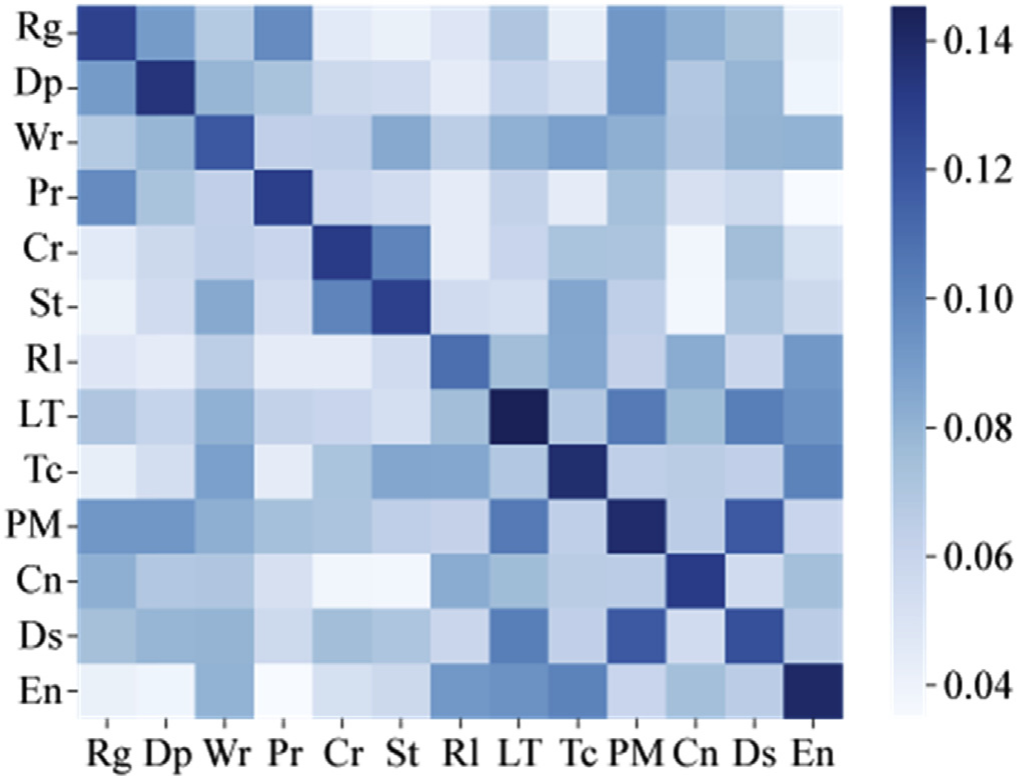
Inter-category meta-data similarity.

Fig. 3 shows all scores of the inter-category meta-data similarities on the causality data. Three combinations of two categories, **Rg**—**Dp**, **Cr**—**St** and **Ds**—**PM** contain more common categories compared to other combinations.

**Fig. 4:**
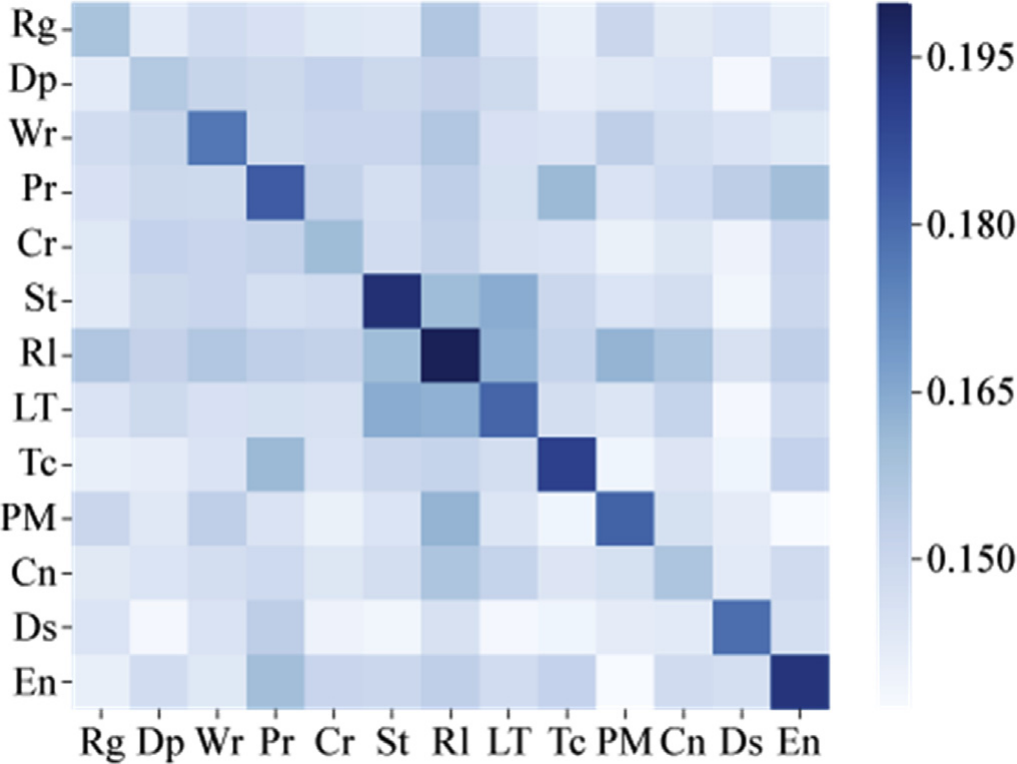
Inter-category MI score.

**Fig. 5:**
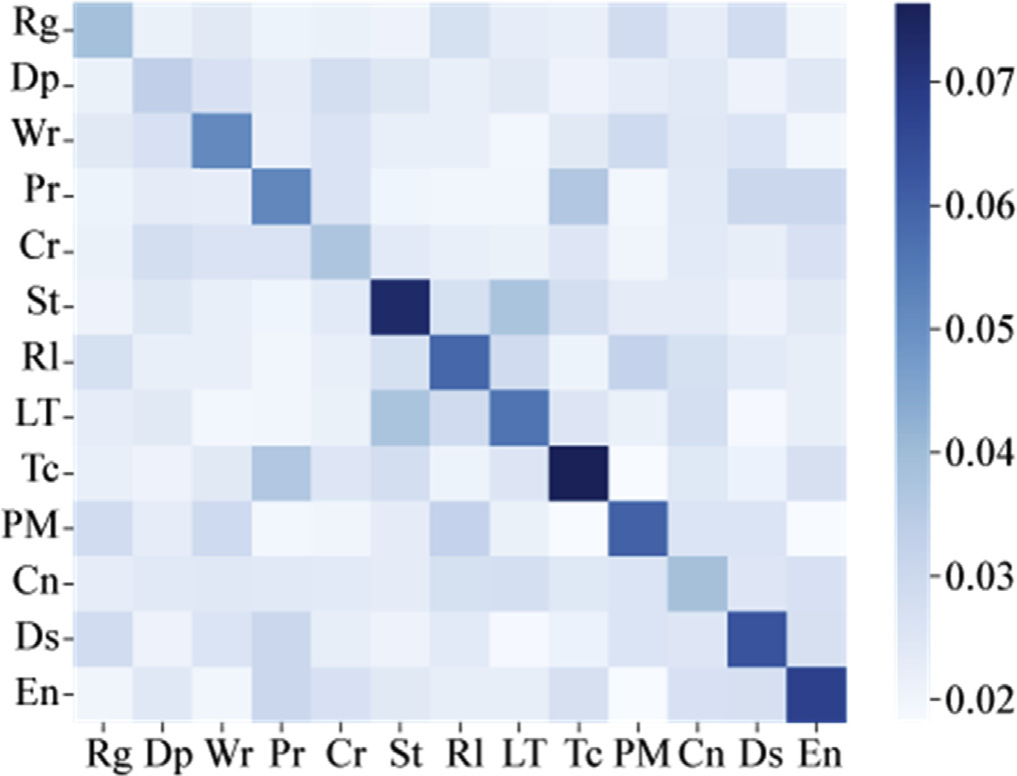
Inter-category Jaccard scores.

**Fig. 6:**
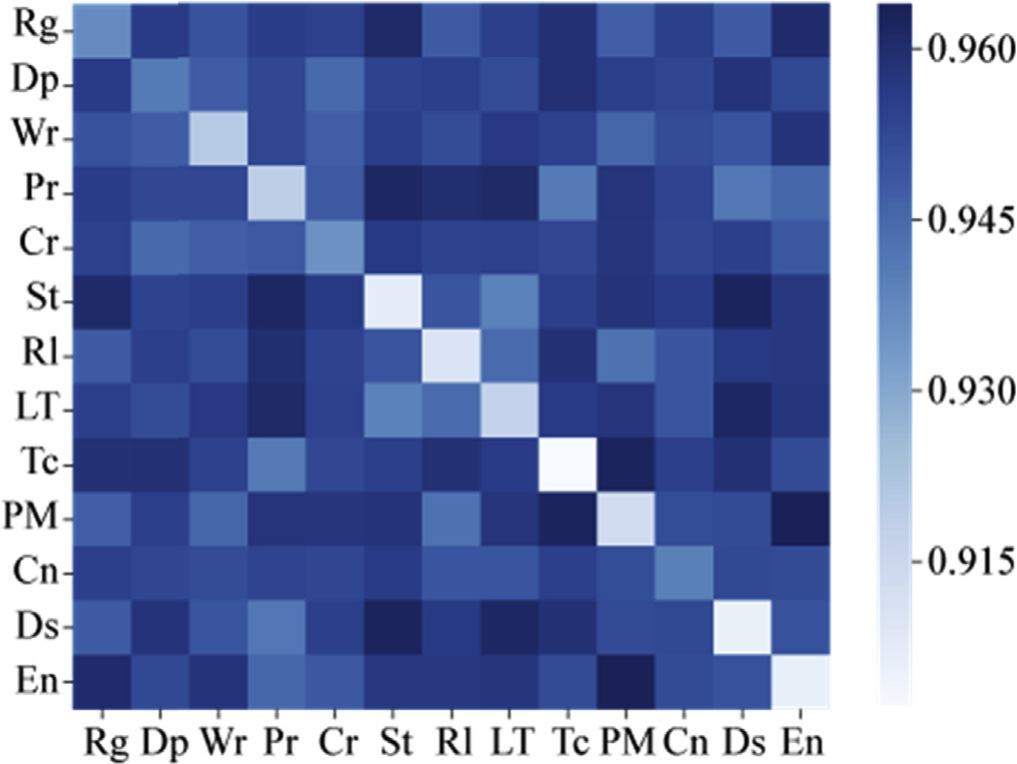
Inter-category TF-IDF + JS scores.

Fig. 4 plots the MI scores for all combinations of categories on the causality data. This figure indicates that three categories (**St**, **Rl** and **LT**) are more similar to each other compared with other category combinations. Fig. 5 plots the Jaccard index for the inter-category analysis. Similar to the scores of MI, three categories (**St**, **Rl** and **LT**) are more similar to each other compared to others. However, the Jaccard similarity scores between the three categories are lower than the MI scores. Fig. 6 shows the TF-IDF + JS scores. Two combinations of categories are similar to each other. The scores for the **St**, **Rl** and **LT** categories are smaller compared to other combinations. In addition, the **Dp**, **Wr**, **Pr** and **Cr** categories have relatively lower scores compared to other combinations except the combination of **St**, **Rl** and **LT**. Thus, these four categories are more similar to each other compared with other category combinations.

### Summary of the Statistical Analyses

All statistical analyses for causalities indicated that the similarities between intra- and inter-category data tended to be low. Thus, if it is necessary to use only past causality data in machine learning study such as IR specialized for history, it is better to use simple techniques, e.g., simple word-based pattern matching and counting common categories. In contrast, if it is able to use both past causality and event data together, using more sophisticated machine learning models such as SVM, naive Bayes classification, and random forests is a good choise as the published data includes 1038 categorized data.

## References

[1] Kimura Y., Sato T., Kishimoto M., Yui O., Aoki K., Komatsu H., Mizushima T., Hashiba Y. (2014). Shosetsu Sekaishi B (Se B 304).

[2] Ogata I., Kawashima S., Goto A., Sakurai Y., Fukui N., Motomura R., Yamamoto H., Nishihama Y. (2014). Sekaishi B (Se B 301).

[3] Ikejiri R., Sumikawa Y. (2020). Raw Data Presented in This Paper.

[4] Ikejiri R., Sumikawa Y. (2016). Developing world history lessons to foster authentic social participation by searching for historical causation in relation to current issues dominating the news. Journal of Educational Research on Social Studies.

[5] Lee P. (2005). Historical literacy: theory and research. International Journal of Historical Learning. Teaching and Research..

[6] Ogata I., Kato T., Kabayama K., Kawakita M., Kishimoto M., Kuroda H., Sato T., Minamizuka S., Yamamoto H. (1994). Encyclopedia of historiography. koubundou.

[7] Calinski T., Harabasz J. (1974). A dendrite method for cluster analysis. Commun. Stat..

[8] Vinh N.X., Epps J., Bailey J. (2009). Information Theoretic Measures for Clusterings Comparison: Is a Correction for Chance Necessary?. https://dl.acm.org/citation.cfm?doid=1553374.1553511.

